# Evaluation of intramural hematoma: a novel use of ^18^F-fluorodeoxyglucose positron emission tomography/magnetic resonance imaging

**DOI:** 10.1186/s13019-024-02598-x

**Published:** 2024-03-15

**Authors:** Fan Yang, Yuanwei Chen, Yongrong Zhou, Dan Shao, Jianfang Luo

**Affiliations:** 1 Department of Emergency and Critical Care Medicine, Guangdong Provincial People’s Hospital, Guangdong Academy of Medical Sciences, Southern Medical University, Yuexiu District, Guangzhou, 83827812-10580 Guangdong China; 2 School of Medicine, South China University of Technology, Guangzhou, Guangdong China; 3 Department of PET Center, Guangdong Provincial People’s Hospital, Guangdong Academy of Medical Sciences, Southern Medical University, Guangzhou, Guangdong China; 4 Department of Cardiology, Guangdong Cardiovascular Institute, Guangdong Provincial Key Laboratory of Coronary Disease, Guangdong Provincial People’s Hospital, Guangdong Academy of Medical Sciences, Southern Medical University, Guangzhou, Guangdong China

**Keywords:** Intramural hematoma, Risk evaluation, ^18^F-FDG PET/MR

## Abstract

**Background:**

Aortic intramural hematoma (IMH) is one of the typical entities of acute aortic syndrome and probably accounts for 5–25% of all cases. The ulcer-like projections (ULP), which are described as a focal, blood-filled pouch protruding into the hematoma of the aortic wall, are regarded as one of the high-risk imaging features of IMH and may cause initial medical treatment failure and death.

**Case presentation:**

We present a case report of an acute type B IMH patient with impaired renal function and newly developed ULP in the acute phase. The ^18^F-fluorodeoxyglucose positron emission tomography/magnetic resonance imaging (^18^F-FDG PET/MR) was performed to evaluate the condition of aortic hematoma. The ^18^F-FDG focal uptake along the aortic wall of the hematoma was normal compared to the background (SUV_max_ 2.17; SUV_SVC_ 1.6; TBR 1.35). We considered the IMH stable in such cases and opted for medical treatment and watchful observation. Six months after discharge, the patient’s recovery was satisfactory, and aortic remodeling was ideal.

**Conclusions:**

The ^18^F-FDG PET/MR is a novel tool to evaluate the risk of IMH patients and thus provides information for therapy selection.

## Background

Acute type B aortic intramural hematoma (IMH) is characterized by dramatic evolution and may evolve into aortic dissection and even rupture, which frequently cause initial medical treatment failure and death [1]. Here, we present a case in which we utilized ^18^F-fluorodeoxyglucose positron emission tomography/magnetic resonance imaging (^18^F-FDG PET/MR) to evaluate the risk of type B IMH.

## Case presentation

**Fig. 1:**
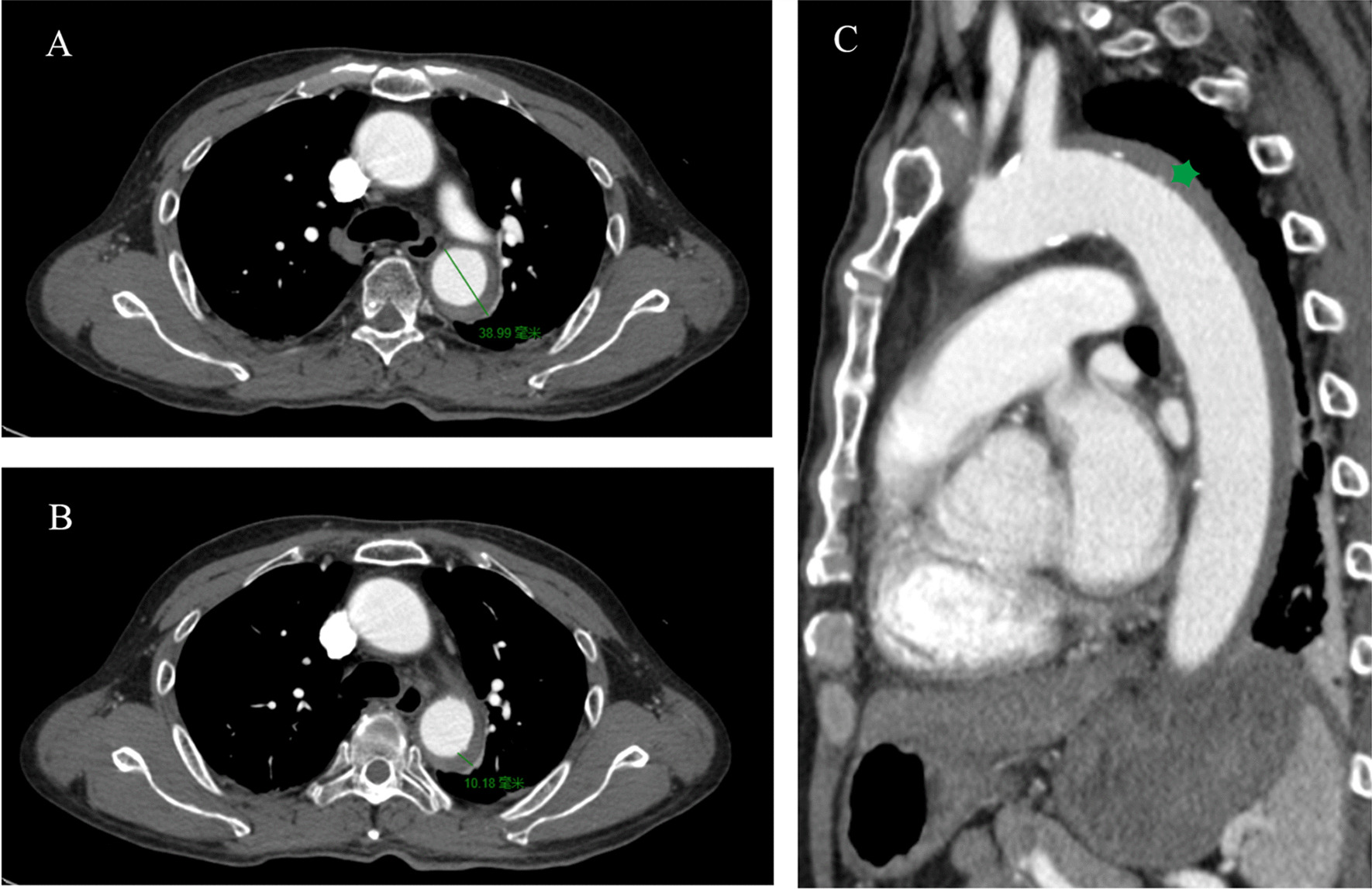
The urgent contrast-enhanced computed tomography image on admission shows intramural hematoma of the descending aorta. **A** The section of the maximal aortic diameter (line). **B** The section of maximal hematoma thickness (line). **C** Sagittal contrast-enhanced computed tomography shows intramural hematoma of the descending aorta (star)

A 58-year-old man presented with severe chest pain and elevated blood pressure (188/79 mm Hg). Heart rate was 120 beats/min and respiratory rate was 22 breaths/min. Oxygen saturation was sufficient on room air. Urgent laboratory testing showed increased creatinine level (185 μmol/L, 2.09 mg/dl) without metabolic acidosis. Urgent contrast-enhanced computed tomography (CT) was performed and an acute type B aortic IMH extending from the distal aortic arch to the iliac artery bifurcation was noted (Fig. 1). The maximal aortic diameter was 39 mm and the maximal hematoma thickness was 10 mm. Initial CT detected no ulcer-like projections (ULP).

**Fig. 2:**
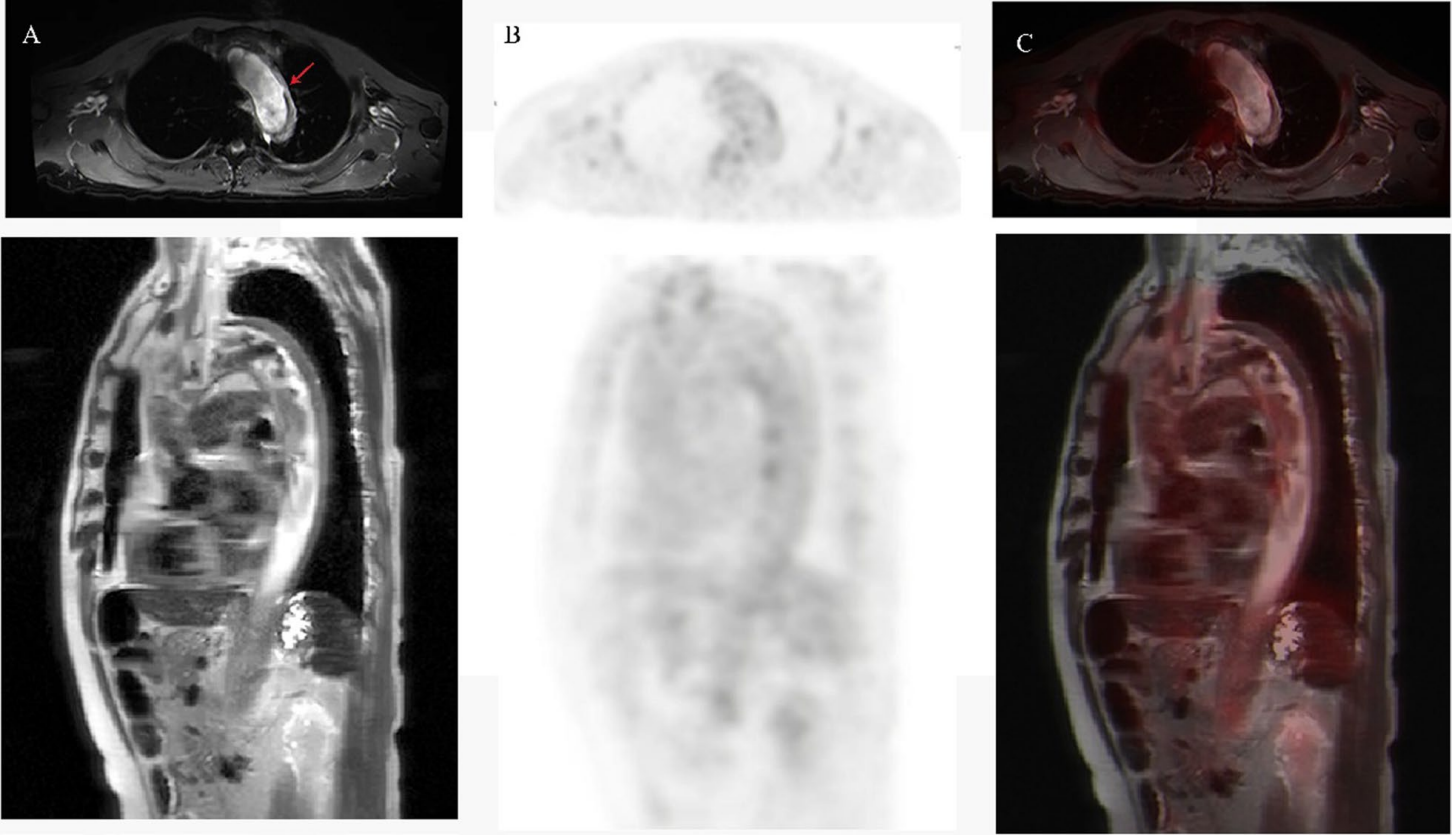
^18^F-fluorodeoxyglucose positron emission tomography/ magnetic resonance imaging. **A** Magnetic resonance imaging shows intramural hematoma with newly developed ULP on the arch (arrow). **B** The metabolism of the aortic wall was assessed by ^18^F-fluorodeoxyglucose positron emission tomography. **C** Fusion image of intramural hematoma

To better evaluate the condition of the aortic hematoma, ^18^F-FDG PET/MR (PET and MRI acquisition parameters can be requested from the authors) was performed three days after admission, and a newly developed ULP located in the distal aortic arch was observed (Fig. 2). The maximum standardized uptake value of the aortic wall (SUV_max_), the maximum standardized uptake value of superior vena cava (SUV_SVC_), and the target-to-blood ratio (TBR, SUV_max_ divided by SUV_SVC_) were calculated through PET. Interestingly, the ^18^F-FDG focal uptake along the aortic wall of the hematoma was normal compared to the background (SUV_max_ 2.17_;_ SUV_SVC_ 1.6_;_ TBR 1.35). The maximal aortic diameter was 36 mm and the maximal hematoma thickness was 9 mm. In such cases, we considered the IMH stable even though it appeared newly developed ULP in the proximal descending aorta. Therefore, we opted for watchful observation and the patient was discharged one week later. Six months after discharge, the patient’s recovery was satisfactory, and aortic remodeling was ideal.

**Fig. 3:**
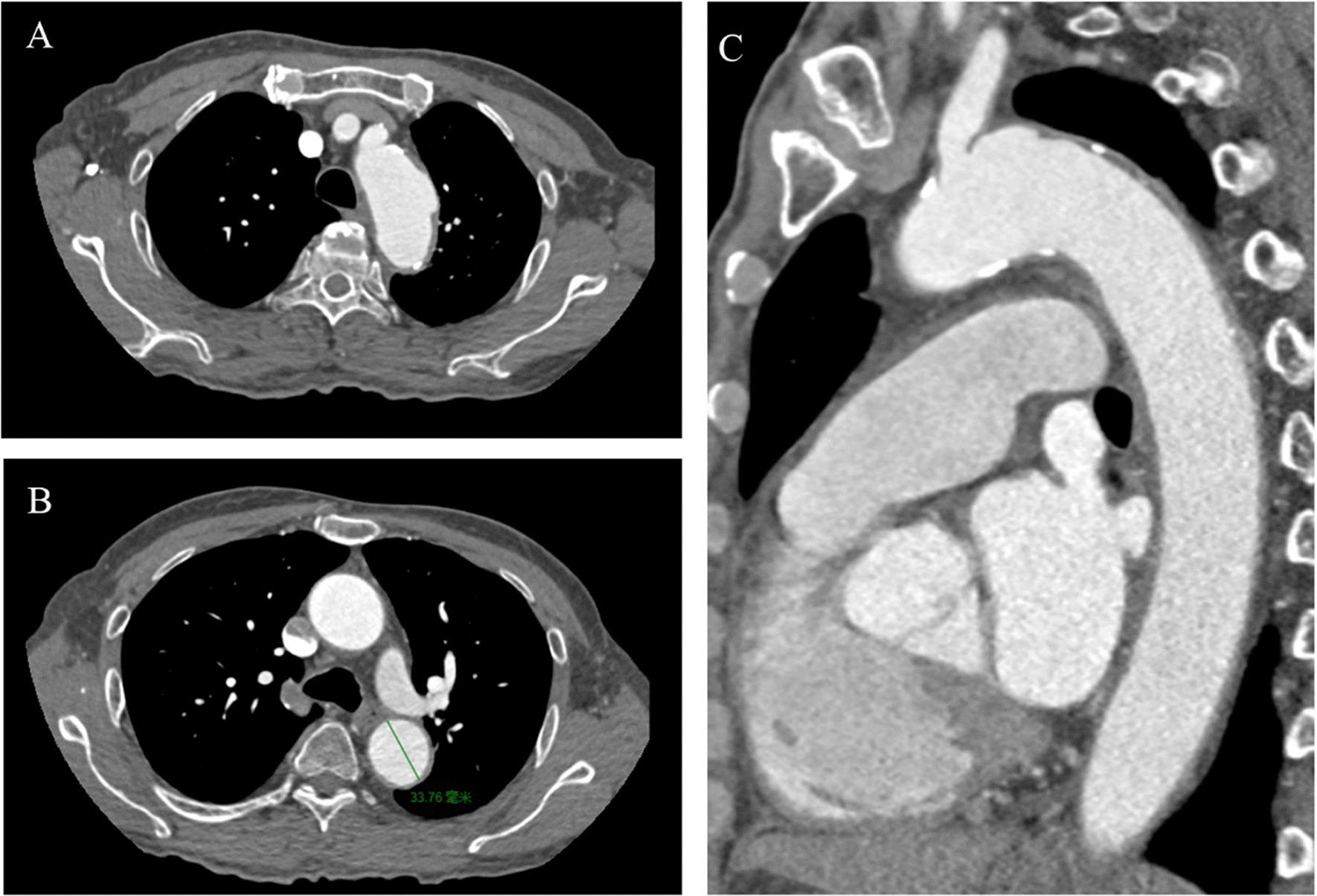
The contrast-enhanced computed tomography image shows the hematoma was significantly resolved and the ULP on the aortic arch disappeared after three months. **A** The transverse section of the aortic arch. **B** The section of the maximal aortic diameter (line). **C** Sagittal contrast-enhanced computed tomography shows resolved intramural hematoma of the descending aorta

The patient’s recovery was satisfactory after six months. He had not experienced chest pain since discharged and the blood pressure (128/75 mm Hg) and heart rate (65–70 bpm) were well controlled. The contrast-enhanced CT showed that, compared with the previous CT result, no matter whether the maximal aortic diameter or the maximal hematoma thickness was reduced (Fig. 3). Owing to the ideal aortic remodeling, the patient received medical treatment continually and was under close follow-up.

## Discussion and conclusions

According to the latest guideline, the ULP involving the descending thoracic aorta if it develops in the acute phase is regarded as one of the high-risk imaging features of IMH [2]. However, in our previous experience, not all forms of acute newly developed ULP are associated with poor outcomes. The ^18^F-FDG PET/CT provided a valuable approach for predicting risk in patients with type B IMH. As for this patient, we performed ^18^F-FDG PET/MR to further assess the IMH condition. It’s known that MR can provide coverage of the entire aorta and branch vessels, which can characterize aortic wall changes in the setting of inflammation and AAS [3]. As mentioned above, the ^18^F-FDG focal uptake along the aortic wall of the hematoma of this patient was normal compared to the background (SUV_max_ 2.17_;_ SUV_SVC_ 1.6_;_ TBR 1.35). Based on our previous findings, the TBR of 1.5 had an acceptable predictive value for differentiating high-risk from low-risk patients. Regarding pathophysiology, acute inflammation could result in the accumulation of hypermetabolic cells such as macrophages and enhanced 18F-FDG uptake [4]. In addition, ^18^F-FDG PET/MR does not require contrast media, which can cause acute kidney injury. Therefore, ^18^F-FDG PET/MR not only evaluated the risk of IMH but also avoided the impairment of this patient's renal function.

This is one of the first case reports of a patient using ^18^F-FDG PET/MR as a complementary diagnostic tool for the anatomic-functional evaluation for patients with high-risk imaging features. Further analysis involving larger samples are suggested to validate the efficacy of ^18^F-FDG PET/MR in IMH patients.

## Data Availability

Data available on request.

## Abbreviations

IMH: Intramural hematoma
AAS: Acute aortic syndrome
ULP: Ulcer-like projections
^18^F-FDG PET/MR: ^18^F-fluorodeoxyglucose positron emission tomography/magnetic resonance imaging
